# Structural Modeling of Protein–DNA Interactions Underlying Genome Copy Number Variation in Nanoviruses

**DOI:** 10.34133/csbj.0156

**Published:** 2026-07-23

**Authors:** Aamir Lal, Myeonghwan Kwak, Muhammad Amir Qureshi, Sukchan Lee, Eui-Joon Kil

**Affiliations:** ^1^ Department of Plant Medicals, Gyeongkuk National University, Andong 36729, Korea.; ^2^ Agricultural Research Institute, Gyeongkuk National University, Andong 36729, Korea.; ^3^ Department of Integrative Biotechnology, Sungkyunkwan University, Suwon 16419, Korea.

## Abstract

Multipartite nanoviruses possess multiple segmented genomes that accumulate at unequal levels during infection, giving rise to dynamic genome formulas. Although the replication of all segments relies on a common replication-associated protein (Rep), the molecular basis underlying segment-specific differences in genome abundance remains poorly understood. Here, we investigated whether protein–DNA interactions at conserved *cis*-regulatory elements are associated with this variability by focusing on the intergenic region (IR) of segment S of milk vetch dwarf virus (MDV). We used a structure-based computational approach to analyze interactions between the MDV S–IR and Rep or movement proteins (MPs) from MDV and faba bean necrotic yellows virus under a consistent computational framework. Molecular docking, molecular dynamics simulations, and molecular mechanics/generalized Born surface area-based interaction energy analyses showed that all complexes formed stable interactions with the MDV S–IR. Δ*G*_bind_ values were broadly comparable across systems, whereas differences in interaction persistence, structural stability, and residue-level contributions were observed between Rep- and MP-containing complexes. In particular, Rep–IR interactions exhibited more consistent and distributed interaction patterns, while MP–IR interactions showed greater variability and more localized contacts. These interaction features were more consistent with previously reported MDV DNA–S abundance trends than docking geometry alone. Together, these findings provide a structural framework to interpret MDV DNA–S abundance patterns in a comparative and hypothesis-generating context rather than as direct evidence of causal regulation.

## Graphical Abstract

**Figure:**
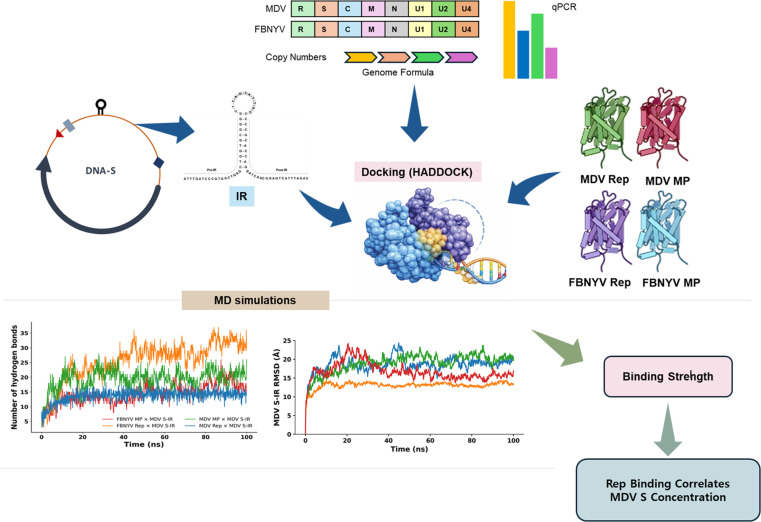

## Introduction

Nanoviruses (family: *Nanoviridae*) are circular single-stranded DNA (ssDNA) viruses characterized by a unique multipartite genome organization, in which the viral genome is divided into multiple distinct segments [1,2]. Each genomic segment is encapsidated separately into individual virions and contains a single open reading frame encoding a protein with a specific function [3,4]. Among these nanovirus genome segments, DNA–R encodes the master replication (M–Rep) initiator protein, which is essential for the rolling-circle replication of all viral genome components [4–7]. DNA–S encodes the capsid protein required for genome encapsidation [3]. DNA–M encodes the movement protein (MP) [1] that facilitates the intercellular transport of the viral genomes, while DNA–C encodes the cell-cycle-link protein [8] that modulates host cell cycle progression to favor viral replication [9]. DNA–N encodes the nuclear shuttle protein that mediates the nuclear export of viral genomes [1,10,11]. DNA–U2 has been recently identified as an RNA silencing suppressor [12], whereas the biological functions of DNA–U1 and DNA–U4 are currently unknown although they are presumed to contribute to viral infectivity [1,2].

**Fig. 1.**
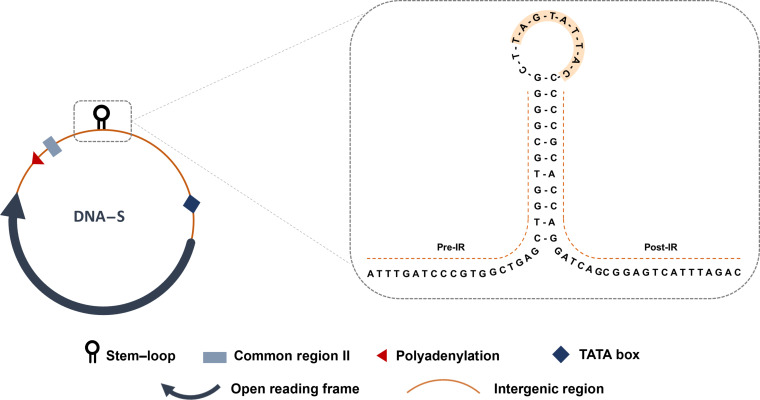
Genome organization and intergenic region architecture of milk vetch dwarf virus (MDV) DNA–S. Schematic representation of the full circular MDV–S genome segment showing the open reading frame (ORF) and the intergenic region (IR). An enlarged view of the MDV S–IR is shown, highlighting the conserved stem–loop structure containing the nonanucleotide motif, together with the flanking pre-IR and post-IR sequences. The pre-IR corresponds to the IR sequences located upstream of the stem–loop, while the post-IR represents the downstream intergenic sequences following the stem–loop; together, these elements constitute the IR.

This multipartite nature of the genome requires precise coordination among all viral components to achieve successful replication and infection [13]. A key factor that enables such coordination is the presence of conserved *cis*-regulatory elements within the intergenic regions (IRs) of each genomic segment [7,14]. These IRs comprise sequence and structural motifs that regulate replication initiation, transcriptional activities, and interactions with viral and host factors [5,7]. Within the IR of each segment, a conserved inverted repeat sequence forms a stem–loop structure within the common region stem–loop (CR–SL) and contains the conserved nonanucleotide sequence TAGTATTAC (Fig. 1) [2,7].

Nanoviruses replicate via a rolling-circle mechanism in which the replication of each genome segment is initiated by the Rep protein, which recognizes and nicks the conserved nonanucleotide sequence [7,15]. Despite the shared replication machinery, genome segments do not accumulate at equivalent levels within infected plants [13,15–17]. Genome formulas based on copy numbers have been analyzed by Sicard *et al.* [18], showing variation in the copy numbers of genomic segments of faba bean necrotic yellows virus (FBNYV). Similarly, measurements of segment copy numbers during milk vetch dwarf virus (MDV) infection revealed substantial differences in the abundance of individual genomic components, resulting in distinct genome formulas rather than a uniform representation of all segments [19].

As replication is intrinsically linked to viral DNA accumulation and given that all nanovirus genome segments rely on Rep-mediated rolling-circle replication, variations in segment copy number are still observed [18,20]. This suggests that replication-associated processes might differ among individual genomic components. One potential level at which such differences could arise is the interaction between the viral Rep protein and conserved *cis*-regulatory DNA elements within the IRs of genomic segments, which serve as the substrate for replication initiation.

To address this gap, we employed a structure-based *in silico* approach to examine protein–DNA interactions in this study, where Rep and MP from MDV and FBNYV were analyzed using a defined ssDNA substrate, the MDV S–IR. Molecular docking, molecular dynamics (MD) simulations and binding free energy (Δ*G*_bind_) analyses were used to examine the stability and dynamics of protein–DNA interactions under a consistent computational framework and to assess whether these interaction features are consistent with experimentally observed differences in genome copy number.

MDV DNA–S was selected as a representative genomic component because experimental analysis of MDV infection has shown that this segment can exhibit pronounced variability and elevated relative abundance [19]. Rather than re-examining genome copy numbers experimentally, we focus on whether the protein–DNA interaction features of the MDV S–IR can offer a structural explanation for previously reported abundance patterns.

## Methods

### DNA substrate and viral protein selection for interaction analyses

All protein–DNA interaction analyses were conducted using the full IR of the MDV DNA–S segment (GenBank accession number: MK726376) as the DNA substrate. This DNA was treated as single stranded in all analyses while preserving internal base-pairing within the IR to reflect its native stem–loop folding. The MDV proteins selected for interaction analysis in this study have been previously reported [19,21,22] and are Rep (GenBank accession number: MK726377) and MP (GenBank accession number: MG852090). For FBNYV (Egyptian isolate) [7], Rep (GenBank accession number: AJ132180) and MP (GenBank accession number: AJ132182) were used. Rep was included due to its established role in recognizing and initiating replication at the IR [5,23], whereas MP was analyzed to evaluate possible DNA-associated interactions that may contribute to viral intercellular movement and accumulation [24].

### Protein structure preparation for interaction analysis

The amino acid sequences of Rep and MP proteins from MDV and FBNYV were retrieved from the sequence databases, as mentioned earlier. Three-dimensional (3D) protein structures were predicted using AlphaFold3, which generates structural models based on the deep-learning-guided prediction of protein folding. For each protein, the top-ranked model was selected based on the internal confidence metrics provided by the prediction pipeline. Model confidence was assessed using predicted local distance difference test (pLDDT) scores, and regions with low confidence were interpreted with caution. The resulting protein models were directly used for subsequent protein–DNA docking analyses.

### Workflow for the 3D structure generation of DNA sequences

**Fig. 2.**
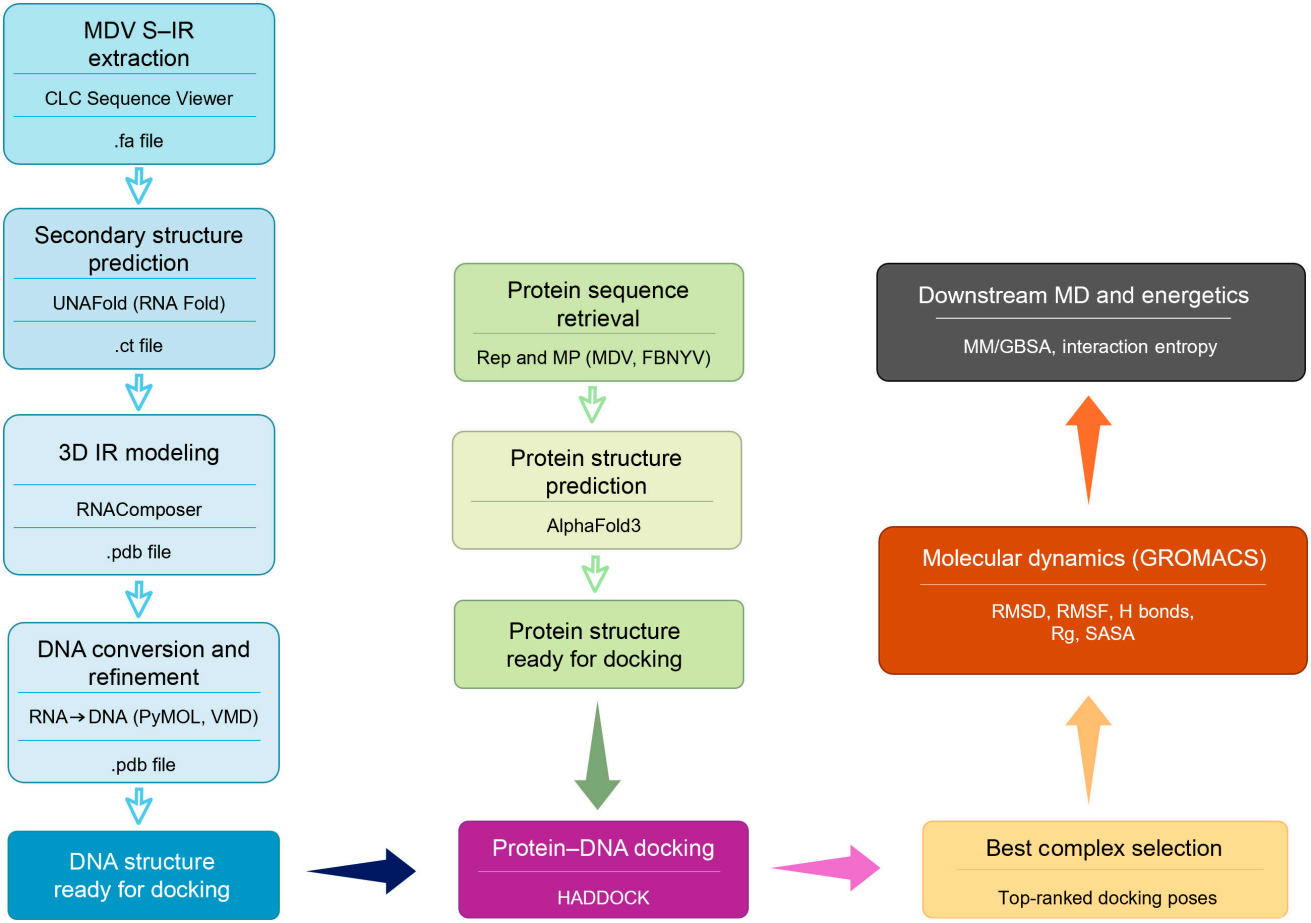
Workflow for protein–DNA interaction analysis involving the milk vetch dwarf virus (MDV) segment S intergenic region (S–IR) in this study. The workflow outlines the sequential steps used to model and analyze interactions between the MDV S–IR and viral replication-associated protein (Rep) or movement protein (MP) from MDV and faba bean necrotic yellows virus (FBNYV). The MDV S–IR sequence was extracted and subjected to secondary structure prediction, followed by 3-dimensional modeling, RNA-to-DNA conversion, and refinement to generate a docking-ready single-stranded DNA (ssDNA) structure. In parallel, Rep and MP protein sequences were retrieved, structurally modeled, and refined to obtain docking-ready protein structures. Protein–DNA docking was performed using HADDOCK, and top-ranked complexes were selected for downstream molecular dynamics (MD) simulations and energetic analyses, including structural stability and Δ*G*_bind_ assessments.

The workflow to construct the 3D structures of the MDV S–IR from the nucleotide sequence consisted of 3 main steps (Fig. 2) and was adapted from a strategy described by Jeddi and Saiz [25], with minor modifications. It involved the generation of the secondary structure of the IR in step 1, followed by the construction of equivalent 3D RNA models in step 2. These single-stranded RNA (ssRNA) models were then translated into 3D ssDNA models in step 3.

### Step 1: Build the ssDNA secondary structure from the sequence

The MDV S–IR sequence was extracted using CLC Sequence Viewer (QIAGEN Aarhus A/S, Aarhus, Denmark) and exported in FASTA format. Secondary structure prediction was performed using the UNAFold web server (RNA Folding Form v2.3) [26] based on free energy minimization. Possible folding conformations were approximated according to Watson–Crick base-pairing, and the most thermodynamically stable structure was selected. The initial sequences were treated as linear molecules and folded at 37 °C with an ionic concentration of 1 M Na^+^ and 0 M Mg^2+^. Only fold configurations within 5% of the minimum free energy were retained, with up to 50 alternative folds generated.

### Step 2: Construct equivalent 3D ssRNA models and refine structures

The predicted secondary structure was used to generate a 3D RNA model. RNAComposer v1.0 (https://rnacomposer.cs.put.poznan.pl/) was applied to translate the secondary structure into a 3D RNA framework. The RNA scaffold preserved the predicted base-pairing while capturing key structural features. The final structure was downloaded in PDB format for downstream analyses.

### Step 3: Transform the 3D ssRNA model into ssDNA 3D structures

The output files of the ssRNA structure in PDB format were first processed in PyMOL (The PyMOL Molecular Graphics System, version 2.2.0, Schrödinger, USA), where uracil residues were renamed to thymine using the command “alter resn U, resn='T'” while preserving the overall 3D fold. The modified structure was then processed in Visual Molecular Dynamics (VMD) [27] using the AutoPSF plug-in together with the CHARMM36 nucleic acid topology file (top_all36_na.rtf), which reassigned atoms according to the updated residue names and converted the ribose sugar to deoxyribose. During this process, missing hydrogen atoms were automatically added to generate a chemically consistent ssDNA structure without altering the original 3D conformation.

To further evaluate the structural reliability of the modeled MDV S–IR, an independent 100-ns MD simulation of the isolated ssDNA stem–loop was performed using the same CHARMM36 force field [28] and simulation protocol applied to the protein–DNA complexes. Structural stability was assessed using backbone root mean square deviation (RMSD), radius of gyration (Rg), stem hydrogen-bond persistence, sugar-pucker analysis, and backbone torsion-angle monitoring standard GROMACS analysis tools [29]. The resulting 3D ssDNA model was used consistently as the docking substrate for all protein–DNA interaction analyses. A representative PyMOL-rendered structure of the final MDV S–IR ssDNA model is provided in Fig. S1 to show the docking substrate used in this study.

### Protein–DNA docking analyses

The refined structures of Rep and MP from both nanoviruses were individually docked with the MDV S–IR (ssDNA structural model) using HADDOCK v2.4 [30]. Docking was performed in a blind manner without defining active or passive residues, allowing unbiased sampling of the protein–DNA interaction interfaces. Each protein–DNA combination was treated as an independent docking analysis resulting in 4 separate docking runs. HADDOCK generated multiple docking models for each protein–DNA pair, which were clustered based on structural similarity. The top-ranked cluster for each system was selected based on the most favorable *z*-score and cluster size. Representative structures from the top clusters were used for downstream MD simulations. While this approach focuses on the most favorable docking solutions, we acknowledge that alternative docking conformations may also represent plausible binding modes and could exhibit stable interactions.

### MD simulations and trajectory analysis

The protein–DNA complex obtained from molecular docking was prepared for MD simulation using CHARMM-GUI [28,31]. The system was parameterized using the CHARMM36 force field, which provides well-validated and widely used parameters for both proteins and nucleic acids. The complex was solvated in a rectangular simulation box with a minimum distance of 10 Å between the solute and the box boundary using the TIP3P water model [32]. The system was neutralized and brought to physiological ionic strength by the addition of KCl ions at a concentration of 0.15 M.

All MD simulations were performed using GROMACS 2023 [29,33]. Prior to production simulation, the solvated system was subjected to energy minimization using the steepest descent algorithm until the maximum force converged below 1,000 kJ/mol/nm. Following minimization, the system underwent a 2-stage equilibration: first under NVT conditions for 100 ps at 303.15 K using the V-rescale thermostat [34], followed by NPT equilibration for 100 ps at 303.15 K and 1 bar pressure using the Parrinello–Rahman barostat [35]. Production MD was subsequently carried out for 100 ns under NPT conditions. Covalent bonds involving hydrogen atoms were constrained using the LINCS algorithm [36], permitting a 2 fs integration time step. Long-range electrostatic interactions were treated using the particle mesh Ewald method [37], and a cutoff of 12 Å was applied for both electrostatic and van der Waals interactions. Trajectory analyses were performed using standard GROMACS tools. The structural stability of the complexes was evaluated using RMSD, calculated after least-squares fitting to the backbone atoms of the complex. In addition, RMSD was analyzed separately for the protein (Cα atoms) and DNA components. Residue-level flexibility was assessed using root mean square fluctuation (RMSF). The compactness of the systems was evaluated using Rg, while solvent exposure was analyzed through solvent-accessible surface area (SASA). Protein–DNA interaction dynamics were further characterized by hydrogen-bond (H-bond) analysis and minimum-distance calculations between the protein and DNA throughout the simulation trajectories.

### Binding free energy calculation

Δ*G*_bind_ calculations were performed using gmx_MMPBSA v1.6 [38] employing the molecular mechanics/generalized Born surface area (MM/GBSA) approach within a single-trajectory framework. Frames were extracted at uniform intervals of 25 frames from the production MD trajectory for analysis. The generalized Born implicit solvent model igb = 5 (Onufriev–Bashford–Case model II) was applied with a salt concentration of 0.15 M, consistent with the explicit solvent simulation conditions. Atomic radii were assigned using the mbondi2 radii set (PBRadii = 4), which is a validated and recommended choice for generalized Born calculations with igb = 5 in CHARMM36-parameterized systems. The temperature was maintained at 303.15 K throughout the analysis, matching the production MD ensemble.

Per-residue energy decomposition was performed using a single-trajectory decomposition scheme to identify key interfacial residues contributing to binding. Decomposition was reported for all residues within 8 Å of the binding interface to capture the complete set of protein–DNA interaction determinants. Results were exported in CSV format for downstream analysis and visualization. Detailed per-residue and frame-wise energy data are provided as Data S1 to S4.

### Integration with genome copy number data

The results from all computational interaction profiles, including docking, MD simulations, and Δ*G*_bind_ analyses, were examined in relation to previously reported genomic abundance patterns of MDV DNA–S. This comparison allowed us to assess whether differences in protein–DNA interaction behavior, particularly in terms of binding stability and energetics, were consistent with variations in MDV DNA–S accumulation.

## Results

### Validation of the isolated MDV S–IR stem–loop structure

To assess the reliability of the modeled DNA structure, the isolated MDV S–IR stem–loop was subjected to a 100-ns MD simulation. The backbone RMSD rapidly converged and remained stable throughout the simulation, with an average value of 0.308 ± 0.015 nm (Fig. S2a). Similarly, the radius of gyration remained essentially constant (1.019 ± 0.011 nm), indicating preservation of the overall stem–loop architecture (Fig. S2b). The stem region retained an average of 10.9 of 13 possible hydrogen bonds during the simulation, supporting maintenance of the predicted secondary structure (Fig. S2c). Analysis of backbone torsion angles and sugar-pucker conformations further demonstrated that the model remained consistent with canonical B-DNA geometry (Figs. S2d and S3). Collectively, these results support the structural stability of the modeled stem–loop prior to protein–DNA interaction analyses.

### Structural features of the MDV S–IR and protein–DNA complexes

**Fig. 3.**
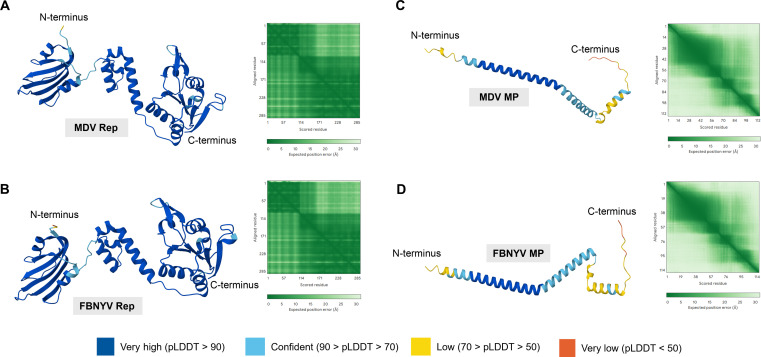
AlphaFold-predicted structures and confidence profiles of replication-associated protein (Rep) and movement protein (MP) from milk vetch dwarf virus (MDV) and faba bean necrotic yellows virus (FBNYV). (A and B) Predicted 3-dimensional structures of Rep proteins from (A) MDV and (B) FBNYV, with N- and C-termini indicated. (C and D) Predicted structures of MPs from (C) MDV and (D) FBNYV. Protein models are colored according to predicted local distance difference test (pLDDT) scores: dark blue (>90) indicates very high confidence, light blue (70 to 90) indicates confident regions, yellow (50 to 70) indicates low confidence, and orange/red (<50) indicates very low confidence. Predicted Aligned Error (PAE) maps are shown adjacent to each structure and represent the expected positional uncertainty between residue pairs. Rep proteins display predominantly high-confidence, well-defined folded regions, whereas MPs exhibit comparatively lower confidence, particularly toward terminal regions, consistent with increased structural flexibility.

The MDV S–IR adopted a compact stem–loop conformation, which is characteristic of nanovirus replication origins. This IR served as a defined ssDNA framework for protein binding. The 3D DNA structure of the IR supported the formation of stable complexes with both Rep and MP proteins from MDV and FBNYV. The structural models of all proteins predicted using AlphaFold showed well-defined folding with high-confidence regions across most of the sequence, with lower confidence primarily confined to the terminal regions (Fig. 3A to D). Notably, MPs exhibited comparatively lower pLDDT scores than Rep proteins, particularly in terminal regions, indicating increased structural flexibility. This pattern is consistent with the elongated and partially flexible architecture observed for MPs in the structural models. The accompanying Predicted Aligned Error maps similarly indicate greater positional uncertainty in these regions, whereas the core structural regions of both Rep and MP proteins were predicted with high confidence.

**Fig. 4.**
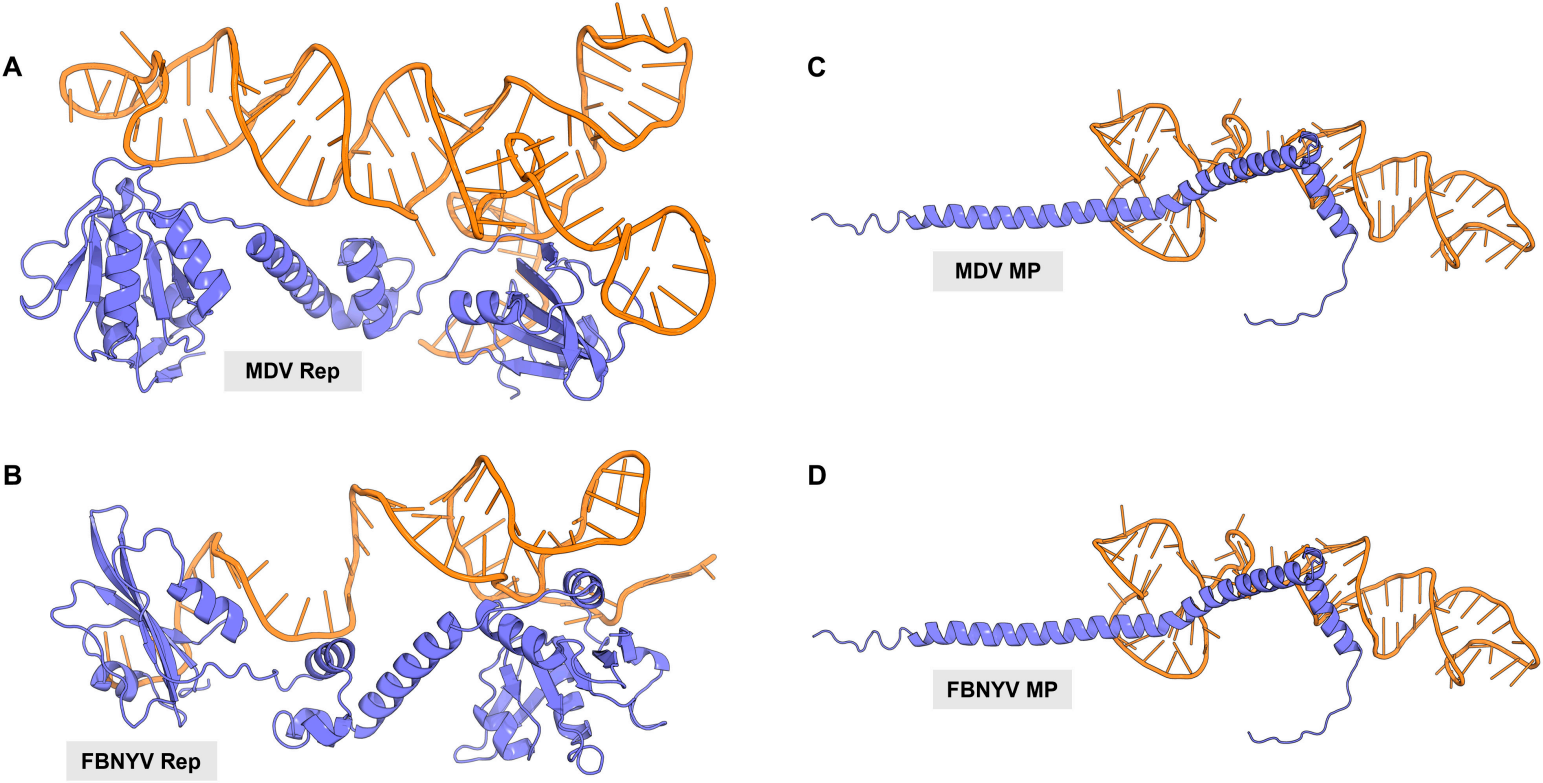
Docking models of replication-associated protein (Rep) and movement protein (MP) from milk vetch dwarf virus (MDV) and faba bean necrotic yellows virus (FBNYV) in complex with the MDV segment S intergenic region (S–IR). (A to D) Representative docking conformations of (A) MDV Rep, (B) FBNYV Rep, (C) MDV MP, and (D) FBNYV MP bound to the MDV S–IR. Proteins are shown in purple (cartoon representation), and the single-stranded DNA (MDV S–IR) is shown in orange. Docking models were generated using HADDOCK, and representative structures from the top-ranked clusters are presented. The MDV S–IR adopts a stem–loop configuration and is positioned along defined regions of the protein surface in all complexes. Panels are displayed in comparable orientations to facilitate visual comparison while preserving structural differences between proteins.

Docking analyses generated 4 protein–DNA complexes with distinct binding orientations (Fig. 4) and interfacial interaction patterns, including hydrogen bonding networks (Fig. S4 and Table 1). In the Rep-containing complexes, both MDV–Rep and FBNYV–Rep formed well-defined interaction interfaces with the stem–loop and adjacent regions of the IR. In both cases, the DNA was positioned within the surface grooves of the proteins, suggesting extensive protein–DNA contacts. Although the complexes are shown in comparable orientations to facilitate visual comparison, intrinsic structural differences between MDV and FBNYV Rep proteins result in distinct domain arrangements and loop conformations (Fig. 4A). In contrast, MPs bound the IR in a more open and less deeply embedded configuration. MDV–MP interacted with a relatively limited region of the DNA, whereas a broader surface of the IR was engaged in the FBNYV–MP complex. In addition, MPs displayed a predominantly linear and extended conformation compared to the more globular architecture of Rep proteins, which may contribute to differences in DNA engagement (Fig. 4B). Although all 4 proteins interacted with the same MDV S–IR, the extent and geometry of DNA contacts differed markedly between Rep and MP, as well as between the MDV and FBNYV proteins.

**Table 1.** HADDOCK-derived docking scores, energetic components, and cluster characteristics of protein–DNA complexes with the MDV S–IR

Parameter	MDV-R	MDV-M	FBNYV-M	FBNYV-R
Total structures clustered	57	52	29	34
Number of clusters	5	5	5	5
Top-ranked cluster	Cluster 3	Cluster 2	Cluster 1	Cluster 1
Cluster size	10	10	12	11
HADDOCK score	−188.4 ± 10.4	−198.4 ± 11.2	−209.1 ± 9.4	−215.3 ± 10.1
*z*-score	−1.5	−1.4	−1.3	−1.4
Van der Waals energy (kcal/mol)	−75.3 ± 4.1	−79.3 ± 5.1	−84.6 ± 4.9	−81.2 ± 6.3
Electrostatic energy (kcal/mol)	−248.7 ± 78.2	−245.7 ± 68.3	−260.4 ± 74.1	−252.1 ± 70.4
Buried surface area (Å^2^)	~2,328	~2,540	~3,081	~2,850

MDV, milk vetch dwarf virus; S–IR, segment S intergenic region; FBNYV, faba bean necrotic yellows virus

### Protein–DNA interface organization and per-residue energetic contributions

**Fig. 5.**
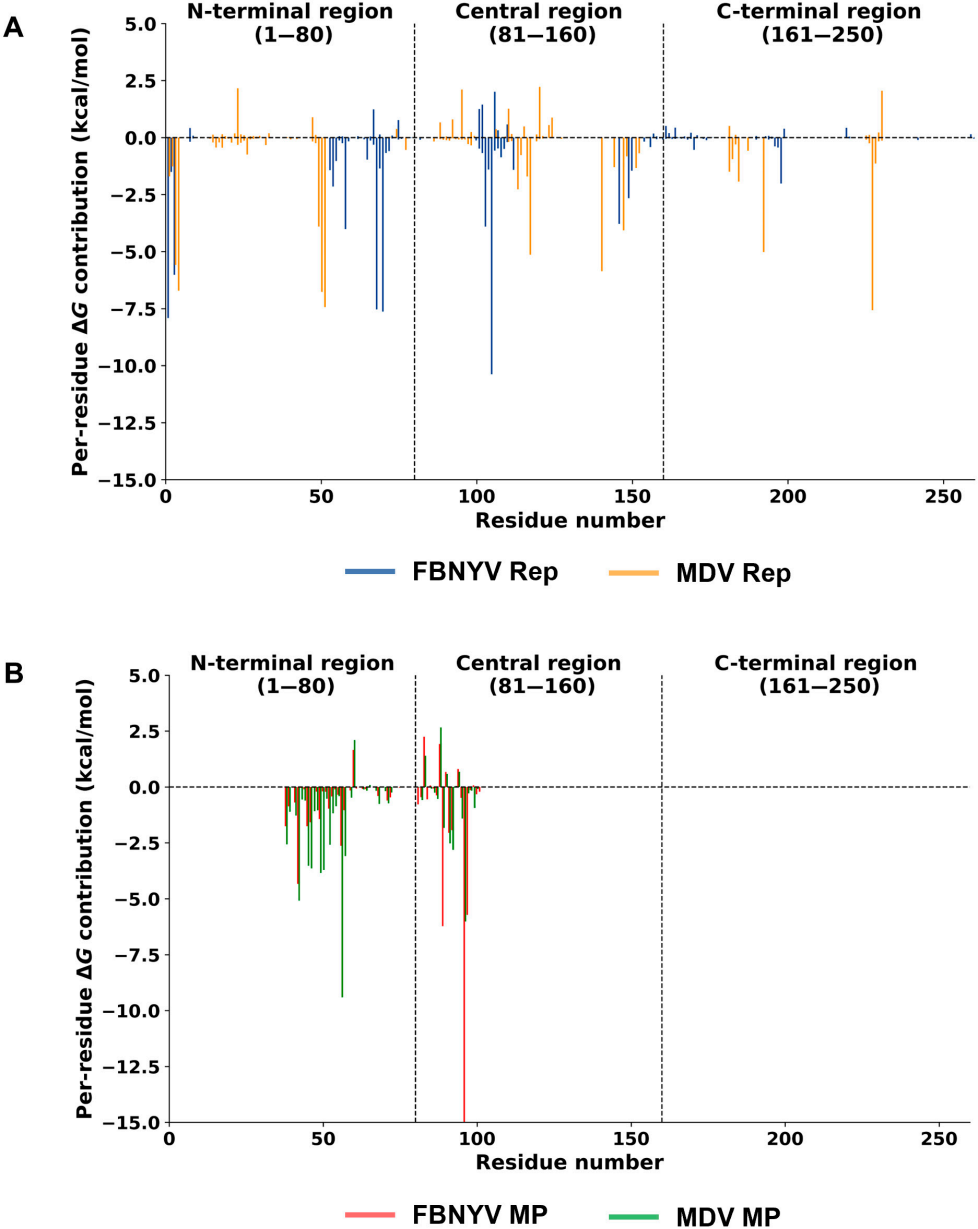
Per-residue binding free energy decomposition of replication-associated protein (Rep)–intergenic region (IR) and movement protein (MP)–IR complexes with the milk vetch dwarf virus (MDV) S–IR. Residue-wise contributions to Δ*G*_bind_ calculated using the molecular mechanics/generalized Born surface area (MM/GBSA) method for (A) Rep–IR and (B) MP–IR complexes. Residues are plotted along the protein sequence, with their corresponding Δ*G* contributions (kcal/mol), where negative values indicate favorable interactions. Residues are grouped into N-terminal (1 to 80), central (81 to 160), and C-terminal (161 to 250) regions, as indicated by dashed vertical lines. Profiles correspond to faba bean necrotic yellows virus (FBNYV) Rep (blue) and MDV Rep (orange) in panel (A), and FBNYV MP (red) and MDV MP (green) in panel (B). Values were derived from per-residue decomposition of the molecular dynamics (MD) trajectories.

The structural integrity of the modeled MDV S–IR was monitored during MD simulations, and the stem–loop architecture remained stable throughout, supporting its suitability for comparative interaction analysis. Inspection of the modeled protein–DNA complexes shows that the MDV S–IR engages a defined and localized surface region of both Rep and MP proteins across homologous and heterologous combinations. Per-residue Δ*G*_bind_ decomposition further revealed that these interactions are not uniformly distributed along the protein sequence but are concentrated within specific regions (Fig. 5 and Fig. S5). In all cases, only a subset of the protein surface is involved in DNA binding, while distal regions of the protein remain spatially separated from the DNA. This indicates that S–IR recognition occurs through specific interaction interfaces rather than through extensive wrapping of the DNA along the protein surface.

Clear differences are observed in how Rep and MP proteins interact with the S–IR. For both MDV– and FBNYV–Rep proteins, strongly favorable energy contributions are predominantly localized within the central region (approximately residues 81 to 160), which corresponds to the location of the stem–loop structure, with additional contributions from the N-terminal region, resulting in a compact and well-defined interface (Fig. 5A). These residues exhibit the most negative Δ*G* contributions, indicating that they form the primary binding interface with the S–IR. Consistent with this energetic pattern, DNA contacts are concentrated within this region, reinforcing the formation of a compact and well-defined interface. Rep proteins show tighter association with the stem–loop region of the S–IR and deeper DNA accommodation, consistent with a more constrained and stable mode of binding.

In contrast, MPs primarily engage the S–IR through their N-terminal regions, with energy contributions distributed across fewer residues and showing greater variability in magnitude. A limited number of residues, particularly near the boundary between the N-terminal and central regions, contribute strongly to binding, while the majority of residues display weak or near-neutral contributions (Fig. 5B). Compared with Rep, MP–DNA interactions are shallower and more variable, with less continuous DNA accommodation and greater flexibility in DNA positioning across complexes. The absence of extended regions of consistently strong Δ*G* contributions in MPs suggests a more dispersed and less structurally constrained binding interface. Thus, while MPs interact with the S–IR through similarly positioned regions along the sequence, their mode of engagement is less focused than that of Rep. Notably, no single residue dominated the interaction in either protein class; instead, binding was supported by the combined contribution of multiple residues across the interface, highlighting a distributed mode of protein–DNA recognition.

### Binding free energy analysis reveals comparable interactions across complexes

**Fig. 6.**
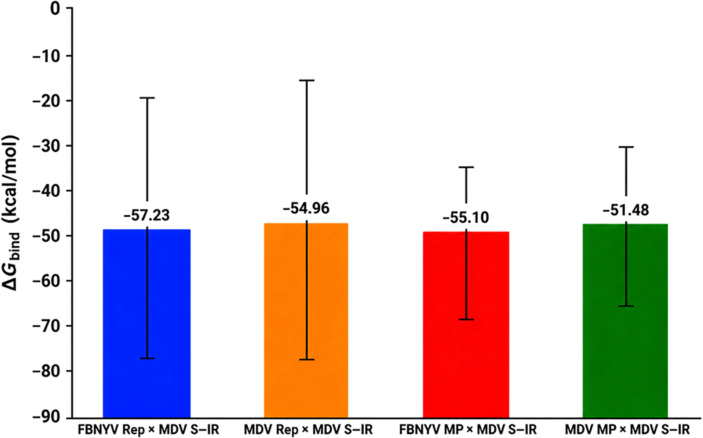
Binding free energy of replication-associated protein (Rep) and movement protein (MP) complexes with the milk vetch dwarf virus (MDV) segment S intergenic region (S–IR). Mean binding free energies (Δ*G*_bind_, kcal/mol) calculated using the molecular mechanics/generalized Born surface area (MM/GBSA) method for faba bean necrotic yellows virus (FBNYV) and MDV Rep and MP proteins in complex with the MDV S–IR. Bars represent the average Δ*G*_bind_ values obtained from molecular dynamics trajectories, and error bars indicate standard deviation. Complexes include FBNYV Rep × MDV S–IR, MDV Rep × MDV S–IR, FBNYV MP × MDV S–IR, and MDV MP × MDV S–IR. Calculations were performed using frames extracted from the production phase of the simulations under identical conditions. Negative values correspond to favorable binding interactions. The calculated values are presented as comparative interaction metrics obtained under identical simulation conditions.

Quantitative Δ*G*_bind_ calculations revealed that all protein–DNA complexes formed energetically favorable interactions with the MDV S–IR (Fig. 6). The MM/GBSA calculations showed that the binding free energies of Rep and MP complexes fall within a relatively narrow range. FBNYV–Rep exhibited the most negative mean Δ*G*_bind_ (−57.23 kcal/mol), followed closely by MDV–Rep (−54.96 kcal/mol), FBNYV–MP (−55.10 kcal/mol), and MDV–MP (−51.48 kcal/mol). However, these differences were small in comparison to the associated standard deviations, indicating substantial variability across the sampled conformations. Because the calculated Δ*G*_bind_ values exhibited substantial overlap in their associated uncertainties, they were interpreted as comparative interaction metrics rather than as rigorous thermodynamic binding free energies. Accordingly, the MM/GBSA results were used primarily as supportive descriptors of interaction behavior and were interpreted together with complementary MD analyses.

### Docking geometry does not directly correspond to binding free energies

The docking scores obtained through HADDOCK v2.4 did not fully align with the binding free energies calculated from MM/GBSA analysis (Table 1). While FBNYV–Rep exhibited the most favorable docking score (−215.3 ± 10.1), followed by FBNYV–MP (−209.1 ± 9.4), MDV–MP (−198.4 ± 11.2), and MDV–Rep (−188.4 ± 10.4), this trend was not directly reflected in the Δ*G*_bind_ values. In contrast to the broader spread observed in docking scores, MM/GBSA calculations showed that all complexes possessed relatively similar binding free energies within a narrow range. This indicates that differences in docking geometry and scoring do not directly translate into differences in binding strength. In the context of MDV S–IR recognition in this study, these observations suggest that docking scores alone are insufficient to evaluate interaction stability in protein–DNA systems and that additional factors such as interaction energetics and conformational behavior should be considered.

### MD analysis reveals distinct stability and interaction patterns of Rep–IR and MP–IR complexes

To evaluate the stability and conformational behavior of Rep–IR and MP–IR complexes, MD simulations were performed for 100 ns under explicit solvent conditions. The structural behavior of the systems was assessed using protein RMSD, RMSF, Rg, SASA, H-bonds, and minimum-distance analyses.

**Fig. 7.**
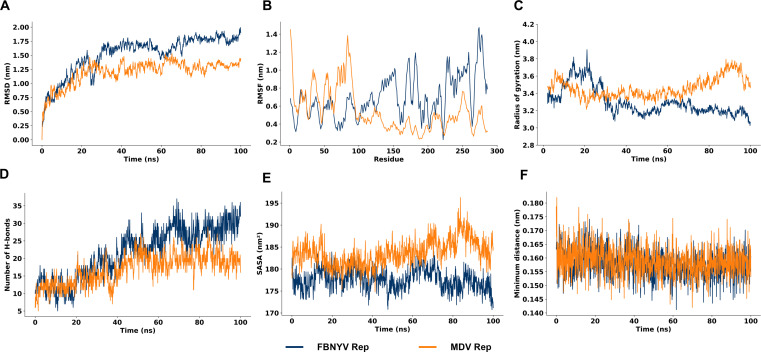
Molecular dynamics (MD) analysis of replication-associated protein (Rep)–intergenic region (IR) complexes formed by faba bean necrotic yellows virus (FBNYV) Rep and milk vetch dwarf virus (MDV) Rep with the MDV S–IR. Structural and interaction parameters monitored during 100-ns MD simulations: (A) root mean square deviation (RMSD) of the protein backbone based on Cα atoms, (B) root mean square fluctuation (RMSF) of protein residues, (C) radius of gyration (Rg), (D) number of protein–DNA H-bonds, (E) solvent-accessible surface area (SASA), and (F) minimum distance between protein and DNA. Trajectories for FBNYV Rep (blue) and MDV Rep (orange) are shown. All metrics were calculated from the production-phase trajectories of the docked protein–DNA complexes under identical simulation conditions.

**Fig. 8.**
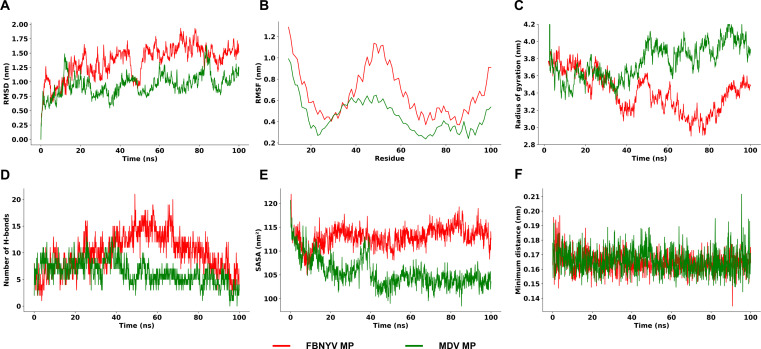
Molecular dynamics (MD) analysis of movement protein (MP)–intergenic region (IR) complexes formed by faba bean necrotic yellows virus (FBNYV) MP and milk vetch dwarf virus (MDV) MP with the MDV S–IR. Structural and interaction properties monitored during 100-ns MD simulations: (A) root mean square deviation (RMSD) of the protein backbone based on Cα atoms, (B) root mean square fluctuation (RMSF) of residues, (C) radius of gyration (Rg), (D) number of protein–DNA H-bonds, (E) solvent-accessible surface area (SASA), and (F) minimum distance between protein and DNA. Profiles for FBNYV MP (red) and MDV MP (green) are shown. All parameters were calculated from the production-phase trajectories of the docked protein–DNA complexes under identical simulation conditions.

The RMSD profiles indicated that all complexes underwent an initial equilibration phase followed by stabilization. In the Rep–IR systems, both FBNYV–Rep and MDV–Rep showed a gradual increase in RMSD during the first 20 to 30 ns before reaching relatively stable trajectories. FBNYV–Rep exhibited slightly higher RMSD values (~1.6 to 1.9 nm) compared with MDV–Rep (~1.2 to 1.4 nm), indicating greater conformational deviation over the trajectory (Fig. 7A). In contrast, MP–IR complexes displayed comparatively lower RMSD values overall. MDV–MP stabilized within ~0.8 to 1.2 nm after the initial equilibration phase, whereas FBNYV–MP showed higher fluctuations (~1.2 to 1.8 nm) throughout the simulation (Fig. 8A). Despite these differences, no abrupt structural deviations were observed in any system. Consistent with these observations, the DNA backbone remained structurally stable throughout the simulations, as indicated by steady RMSD profiles and moderate residue-level fluctuations (Fig. S6). RMSF revealed distinct fluctuation patterns between Rep and MP proteins (Figs. 7B and 8B). Rep proteins showed elevated fluctuations across several regions, particularly toward the C-terminal half in FBNYV–Rep, whereas MDV–Rep exhibited comparatively lower and more localized fluctuations. In MPs, fluctuations were generally distributed along the sequence, with FBNYV–MP showing higher amplitude variations compared with MDV–MP, especially in central regions.

Rg analysis showed that all complexes remained structurally compact throughout the simulations (Figs. 7C and 8C). Rep–IR systems exhibited gradual stabilization, with FBNYV–Rep maintaining slightly higher Rg values (~3.3 to 3.6 nm) than MDV–Rep (~3.1 to 3.3 nm). In contrast, MP–IR complexes showed more pronounced variability, with MDV–MP exhibiting higher Rg values (~3.6 to 4.1 nm) compared with FBNYV–MP (~3.0 to 3.6 nm), indicating differences in overall compactness between the 2 MP systems.

Hydrogen-bond analysis further highlighted differences in interaction patterns between Rep–IR and MP–IR complexes (Figs. 7D and 8D). Rep-containing systems formed a higher number of protein–DNA H-bonds over the trajectory, with FBNYV–Rep consistently exhibiting greater H-bond counts (~20 to 35) compared to MDV–Rep (~15 to 25), indicating a more extensive interaction network. In contrast, MP–IR complexes displayed fewer H-bonds overall, fluctuating within a lower range (~5 to 15), consistent with reduced interaction density at the protein–DNA interface. To further characterize interaction stability, additional analyses of hydrogen-bond geometry and temporal behavior were performed (Figs. S7 and S8). Donor–acceptor distance distributions exhibited a sharp peak around ~0.27 nm with negligible contributions beyond ~0.35 nm, while donor–hydrogen–acceptor angle distributions remained within typical ranges for protein–DNA H-bonds, confirming geometrically favorable interactions. Temporal persistence analysis using hydrogen-bond autocorrelation functions revealed a rapid initial decay followed by a slower relaxation phase in all systems, indicating the coexistence of short-lived and more persistent H-bonds at the interface. Notably, the FBNYV–Rep complex exhibited a comparatively slower decay profile, suggesting a greater proportion of persistent interactions relative to the MDV–Rep system. Together, these results indicate that while H-bonds are dynamically formed and disrupted during the simulation, they collectively contribute to a stable and continuously maintained protein–DNA interface.

SASA analysis showed that Rep–IR complexes maintained relatively stable solvent exposure, with minor fluctuations over time (Fig. 7E). In comparison, MP–IR systems exhibited broader variations in SASA, particularly in FBNYV–MP, reflecting differences in surface exposure during the simulation (Fig. 8E).

Minimum-distance analysis indicated that all complexes maintained consistent protein–DNA proximity throughout the simulations, with distances remaining within a narrow range (~0.15 to 0.17 nm) and no evidence of dissociation events (Figs. 7F and 8F). Overall, these results highlight more consistent interaction patterns in Rep–IR complexes, whereas MP–IR complexes display greater structural variability across the simulation. Among the 4 systems, FBNYV–Rep showed the most persistent interaction pattern based on higher H-bond counts, whereas MDV–MP displayed the least interaction, with fewer and more variable contacts.

### Computational interaction profiles provide structural context for MDV DNA–S abundance patterns

After comparing all parameters of computational analysis across the 4 protein–DNA interaction scenarios, we observed that differences in interaction patterns and stability were broadly consistent with previously reported trends in MDV DNA–S abundance. In the context of MDV infection, Rep–IR interactions displayed more consistent and persistent interaction behavior compared with MP–IR interactions, as reflected in H-bond patterns and structural stability during MD simulations. In contrast, MP–IR complexes showed comparatively higher variability in interaction profiles. Taken together, these observations provide a structural context in which differences in interaction persistence and stability, rather than overall Δ*G*_bind_ alone, may be associated with the observed variability in MDV DNA–S abundance.

## Discussion

Multipartite nanoviruses possess a unique genomic architecture in which the viral genome is divided into multiple, separately encapsidated segments [1,2]. To ensure the successful infection coordinated delivery, replication, and expression of all essential genome components within the same host are required [4,13,39]. Despite this requirement for coordination, the relative abundance of individual genome segments is not fixed during infection. Variation in segment copy number depends on multiple factors, including host species, host-associated factors, and vector transmission dynamics. These variations result in dynamic genome formulas rather than a conserved stoichiometric pattern [16,18]. This indicates that although the presence of genome segments is sufficient to sustain infection, their relative abundance is not strictly conserved [18,19]. Understanding the molecular basis of this variability remains a central question in multipartite virus biology, and here we address one defined molecular dimension of this problem by focusing on the MDV–S IR as a common interaction substrate.

Among all MDV genome segments, DNA–S has been reported to exhibit pronounced variability in abundance during infection [19]. In our recent reassortment experiments examining functional complementation between MDV and FBNYV, we observed that the copy number of the DNA–S segment showed pronounced variation across different reassortant combinations. These observations suggest that DNA–S accumulation may be influenced by molecular processes involved in replication and genome regulation. This motivated us to examine whether intrinsic properties of the MDV S–IR could contribute to such variability at the level of protein–DNA interactions. As Rep is responsible for replication initiation [5,7], variation in Rep–IR interaction behavior represents a plausible molecular factor contributing to segment-specific differences in copy number [23,40]. However, it remains unclear whether Rep engages *cis*-regulatory elements with similar efficiency across homologous and heterologous contexts. To address this gap, we selected the MDV S–IR as a common DNA substrate to enable direct comparison of protein–DNA interactions. Both homologous and heterologous viral proteins were analyzed under a consistent simulation framework, allowing direct comparison across systems. Importantly, the full IR sequence, including the stem–loop and flanking regions, was retained to preserve all known *cis*-acting regulatory elements [41]. This also ensured that the DNA substrate remained representative of the native regulatory features involved in replication and genome regulation. This approach is supported by previous studies demonstrating that IRs function as multifunctional regulatory platforms containing *cis* elements that independently influence replication and transcriptional output of individual genome segments [14,41–45].

In addition to the Rep proteins of MDV and FBNYV, we also used MPs from both MDV and FBNYV in this analysis. MPs are best known for their role in intracellular and intercellular transport of viral genomes [1,24,46]. Although MPs are not directly involved in replication initiation, they may contribute indirectly to genome accumulation by influencing genome trafficking, spatial distribution, and availability at replication sites [40,47,48]. By including MPs in this analysis, we were able to distinguish replication-centered interactions from those related to genome movement. This comparative approach also enabled us to evaluate the differential compatibility of non-Rep proteins with the MDV S–IR.

Recent computational studies have shown that integrating sequence and structural information improves the prediction of protein–nucleic acid interactions. For example, DeepFusion incorporates RNA sequences and experimentally derived structural information to enhance protein–RNA interaction prediction [49]. Similarly, recent end-to-end machine learning frameworks have been developed to predict RNA–protein interactions from sequence-derived features [50,51], highlighting the growing role of computational approaches in nucleic acid–protein interaction studies. In contrast, the present study employs molecular docking and MD simulations to examine protein–DNA interaction stability and residue-level organization. Although the ssDNA model was derived from an RNA-based template, the independent 100-ns MD simulation of the isolated MDV S–IR supported the stability of the modeled stem–loop structure (Figs. S2 and S3). The maintenance of overall compactness, stem hydrogen-bond persistence, and DNA-like backbone conformational behavior provides additional confidence in its use as a comparative docking substrate for protein–DNA interaction analysis. During MD simulations, all protein–DNA complexes, including both homologous and heterologous combinations, maintained stable associations with the MDV S–IR throughout the 100-ns trajectories. However, clear differences in stability and interaction behavior were observed between Rep–IR and MP–IR systems (Figs. 7 and 8). Rep-containing complexes showed more consistent structural behavior, characterized by gradual equilibration, sustained compactness, and higher numbers of protein–DNA H-bonds, whereas MP-containing complexes exhibited greater conformational variability and reduced interaction density. Among all systems, the FBNYV–Rep complex displayed the most stable and persistent interaction profile, while the MDV–MP complex showed the least stable behavior, consistent with its lower interaction density and higher structural variability. Despite these differences, none of the systems showed dissociation or loss of DNA contact, indicating that cross-species protein–DNA compatibility is structurally feasible. These findings are consistent with experimental observations demonstrating that nanovirus proteins can functionally complement across species even when genomic segments are exchanged [7].

The MDV S–IR consistently contacted regions biased toward the N-terminal half of both Rep and MP proteins, and this general binding orientation was maintained across homologous and heterologous protein–DNA complexes (Fig. 5). For Rep proteins, the dominant interaction interface is centered in a structured central region biased toward the N-terminal half, whereas MPs engage the S–IR primarily through N-terminal regions with interaction contributions that are localized and discontinuous along the sequence. These results are in line with previous studies showing that Rep DNA-binding activity and nucleic acid-associated regions of MPs are concentrated toward their N-terminal domains in related plant viruses [52–56]. Although full-length nanovirus Rep–IR or MP–DNA complex structures are not currently available, studies on related ssDNA viruses have shown that Rep proteins recognize origin stem–loop DNA through conserved domains. In geminivirus Rep proteins, the N-terminal region is crucial for origin recognition and DNA cleavage and nucleotidyl transfer [57]. Together, these observations suggest that S–IR binding relies on shared structural features and domain organization rather than strict sequence identity, with Rep proteins forming a more continuous interaction surface and MPs exhibiting a more restricted interaction footprint. These observations support the idea that conserved intergenic architecture can interact with multiple viral proteins across species [41,58–60]. In the case of Rep, engagement of the S–IR through a central, N-terminal-biased interface particularly around the stem–loop region is consistent with its role in replication initiation, where specific recognition and binding of the stem–loop structure is required. In contrast, MP is not a replication initiator [24] and associates with the MDV S–IR through fewer energetically important residues, resulting in a more localized and variable interaction pattern. This supports a more transient and structurally flexible mode of DNA association for MPs. Per-residue energy decomposition analysis also reveals that Rep complexes exhibit stronger and more consistent residue-level contributions compared to MP systems (Fig. S5) [61].

We also observed differences in interaction strength and dynamics between MDV– and FBNYV–Rep proteins in complex with the MDV S–IR (Fig. 6). Although the MM/GBSA-derived interaction energies were broadly comparable across systems, complementary MD analyses revealed differences in interaction behavior, particularly in contact persistence, hydrogen-bonding patterns, and residue-level interaction profiles. The MM/GBSA-derived interaction energies should be interpreted cautiously, as single-trajectory approaches may be influenced by conformational flexibility and associated uncertainties, particularly in protein–DNA systems. Therefore, the calculated Δ*G*_bind_ values were used primarily as comparative descriptors and interpreted together with complementary metrics such as hydrogen-bond persistence, minimum-distance measurements, residue-level interaction profiles, and overall MD stability. Taken together, these observations suggest that differences in Rep–IR interaction behavior may contribute to variation in replication-associated processes and provide a structural context for previously reported differences in segment abundance. Importantly, similar concepts have been identified in other rolling-circle replicating plant viruses, where disruption of Rep–origin interactions does not necessarily abolish replication but can limit genome accumulation and competitive fitness [61,62]. Thus, Rep–IR interaction quality appears to influence replication efficiency rather than serve as a strict on/off determinant. In contrast, MP–IR interaction profiles suggest a more flexible and indirect mode of DNA engagement. This is consistent with the primary role of MPs in genome transport and positioning, rather than direct involvement in replication-driven segment amplification [24,46,48].

We attempted to relate the computational interaction profiles to previously reported trends in MDV DNA–S abundance and found that differences in interaction behavior, particularly in terms of stability and persistence observed during MD simulations, were more consistent with these trends than docking geometry alone. In contrast to docking scores, which showed a broader spread across complexes, binding free energies were relatively similar, indicating that total Δ*G*_bind_ alone does not fully explain differences in interaction profiles. These results highlight that docking scores do not reliably capture the dynamic and structural features associated with protein–DNA interactions when considered in isolation. Although docking is effective for identifying plausible binding orientations, it does not capture important factors such as interaction persistence, conformational adaptability, and residue-level contributions that emerge during MD simulations [63,64]. An additional limitation of this study is that MD simulations were initiated from representative top-ranked docking conformations. Although these complexes remained stable throughout the simulations, alternative binding modes may also exist and could exhibit different interaction characteristics. Furthermore, all analyses were performed using a single DNA substrate, the MDV S–IR, which was selected because of its biological relevance and its association with the DNA–S segment that exhibits pronounced variation in abundance during MDV infection and experimental reassortment studies. While the use of a common substrate enabled direct comparison of homologous and heterologous protein interactions under identical conditions, the observed interaction patterns may be influenced by the specific sequence and structural features of the MDV S–IR. Consequently, the findings should not be assumed to represent all nanovirus genomic segments or related viruses. Future studies incorporating additional IRs from MDV and other nanoviruses will be required to determine the extent to which these interaction patterns are conserved across different genomic contexts. Together, these findings suggest that dynamic features of protein–DNA engagement may provide a structural context for interpreting MDV DNA–S abundance patterns. However, because genome abundance is also influenced by host factors, replication kinetics, segment compatibility, and viral movement, these computational findings should be interpreted as hypothesis generating rather than as direct evidence of causal regulation.

## Conclusion

Our study provides structural insight into how protein–DNA interaction behavior may contribute to variations in genome segment abundance in multipartite nanoviruses. Analysis of homologous and heterologous Rep and MP interactions with the MDV S–IR under identical conditions showed that the conserved IR architecture supports protein binding across different viral backgrounds. However, differences in interaction organization, persistence, and stability observed during MD simulations may represent one contributing factor to genome segment abundance and replication efficiency [65].

To our knowledge, this work represents one of the first structure-based computational analyses integrating docking, MD simulations, and MM/GBSA-based energetics to examine protein–DNA interactions in nanoviruses. This study provides a structural framework for interpreting segment-specific genome accumulation based on interaction behavior rather than binding affinity alone. Although our study relies on *in silico* modeling and simulations and therefore cannot capture the full complexity of viral replication in plants, the interaction patterns we observed are broadly consistent with trends seen in genome copy number data. At the same time, it is important to emphasize that protein–DNA interaction behavior alone is unlikely to fully determine genome segment abundance or replication efficiency. Additional aspects such as host factors, host defense responses, interactions among viral genome segments, and the distinct functional roles of individual segments were not addressed here and may substantially influence infection outcomes. Accordingly, the results presented here should be viewed as describing one contributing molecular dimension rather than a complete explanation. Overall, our results provide a structural perspective on genome segment regulation that emphasizes interaction persistence and organization and offer a foundation for future studies that explore these interactions alongside additional host- and virus-level regulatory processes.

## Data Availability

All data supporting this study are available within the article and its supplementary materials. Additional per-residue and frame-wise energy datasets are provided as Data S1 to S4.
