# Supplementary material for: Structural Modeling of Protein–DNA Interactions Underlying Genome Copy Number Variation in Nanoviruses
Source: Comput Struct Biotechnol J. 2026 Jul 23;35(1):0156. doi: 10.34133/csbj.0156 (PMC13392288; doi:10.34133/csbj.0156)
Supplement: Supplementary 1 — Figs. S1 to S8 Data S1 to S4 [file csbj.0156.f1.zip › Supplementary Information.pdf]

# Structural modeling of protein–DNA interactions underlying genome copy number regulation variation in nanoviruses

Aamir Lal[1,2], Myeonghwan Kwak[1,2], Muhammad Amir Qureshi[1,2], Sukchan Lee[3] and Eui-Joon Kil[1,2,*]

[1]Department of Plant Medicals, Gyeongkuk National University, Andong 36729, Korea

[2]Agricultural Research Institute, Gyeongkuk National University, Andong, 36729, Korea

[3]Department of Integrative Biotechnology, Sungkyunkwan University, Suwon 16419, Korea

## Supplementary Information

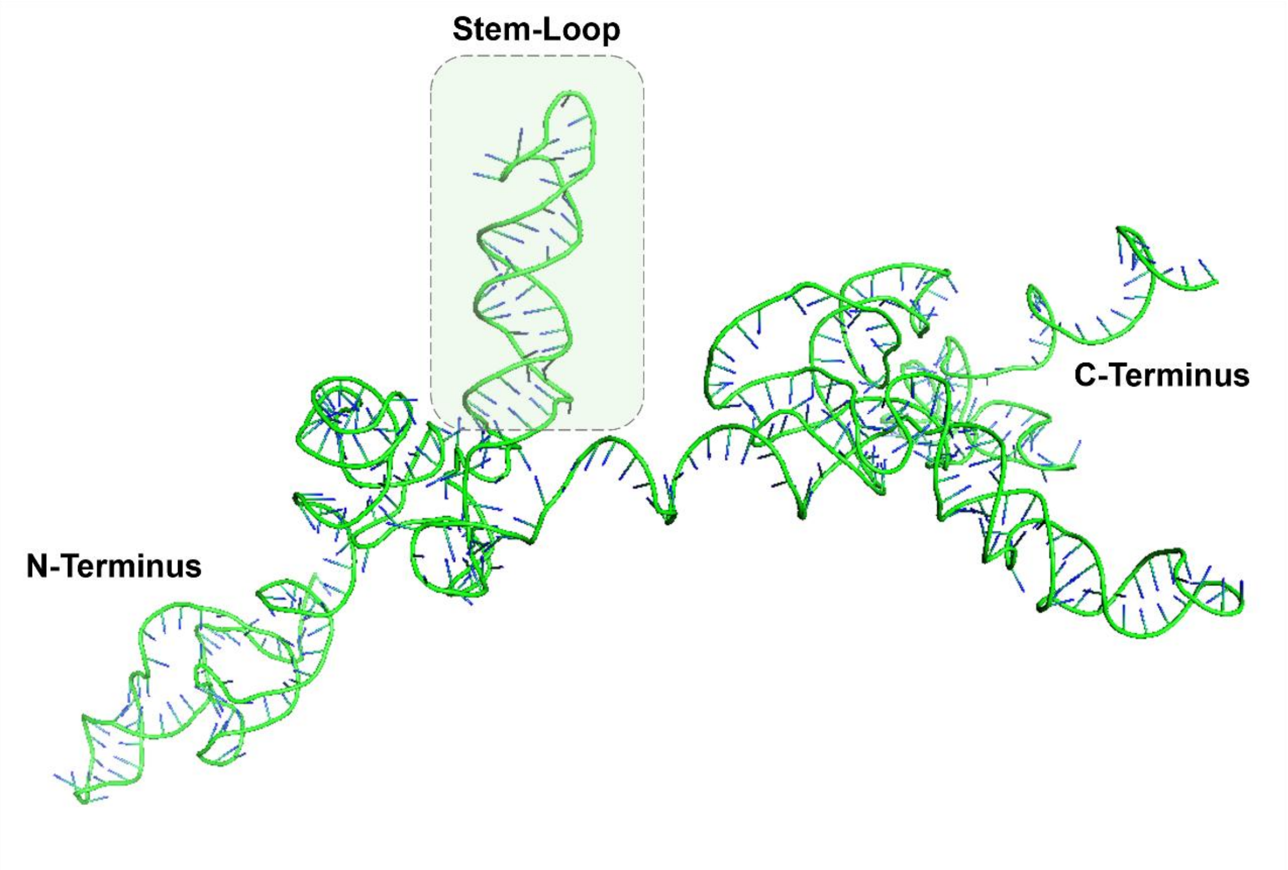


**Figure S1. Three-dimensional structural model of the MDV S–IR single-stranded DNA highlighting the predicted stem–loop architecture.** The model was generated based on secondary structure prediction followed by three-dimensional reconstruction and refinement. The stem–loop region is indicated in shade d box The N- and C-terminal ends of the ssDNA are labeled to illustrate overall topology and orientation. The structure demonstrates a stable folded conformation with a well-defined hairpin, which serves as the binding substrate for subsequent docking and molecular dynamics simulations.

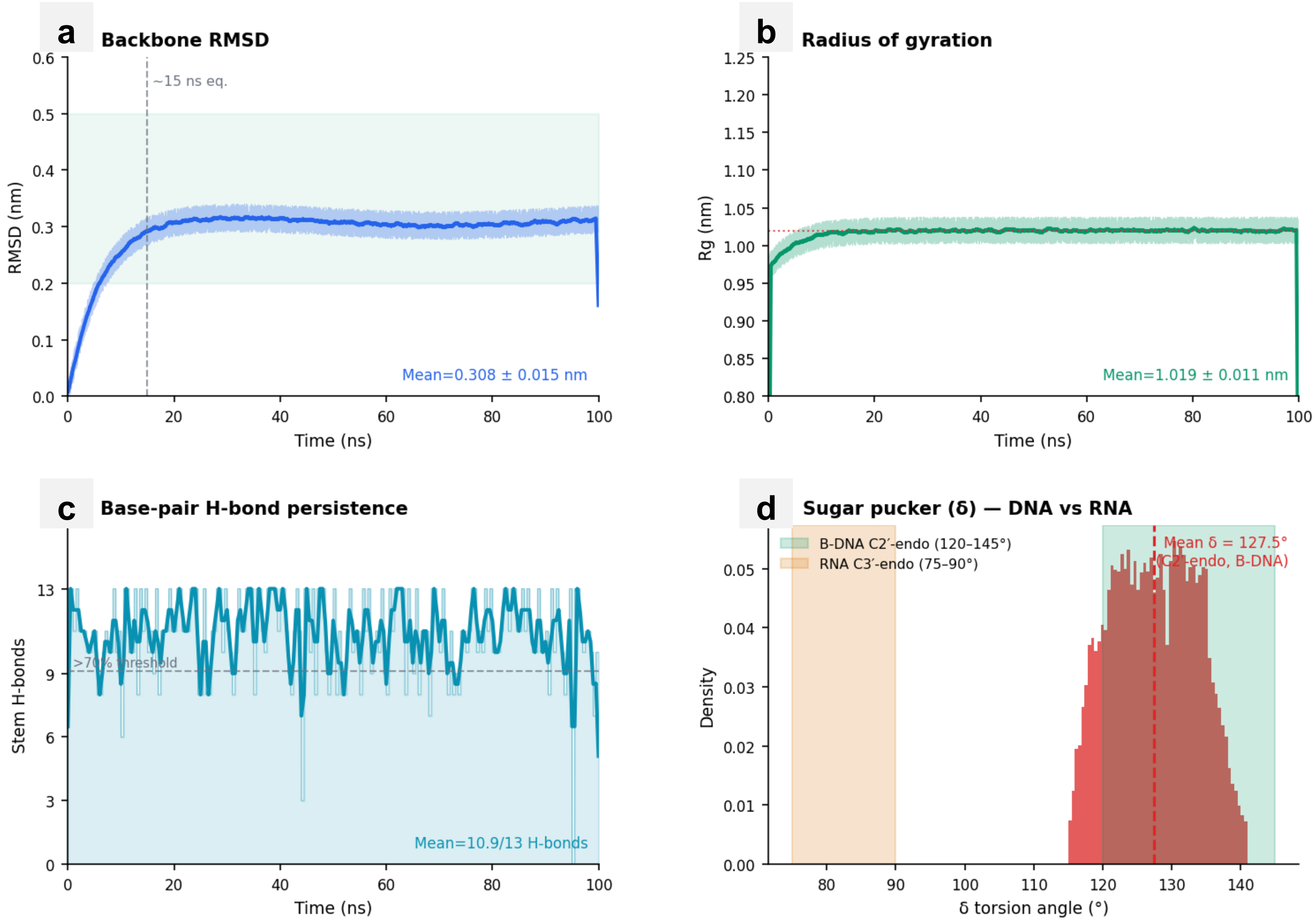


**Figure S2. Validation of the isolated MDV S–IR ssDNA stem–loop during a 100 ns molecular dynamics simulation.** (a) Backbone RMSD. (b) Radius of gyration (Rg). (c) Stem base-pair hydrogen-bond persistence. (d) Sugar-pucker (δ) distribution showing predominant occupancy within the canonical B-DNA C2′-endo conformational range. Collectively, these analyses support the structural stability of the modeled MDV S–IR used in subsequent protein–DNA interaction studies.

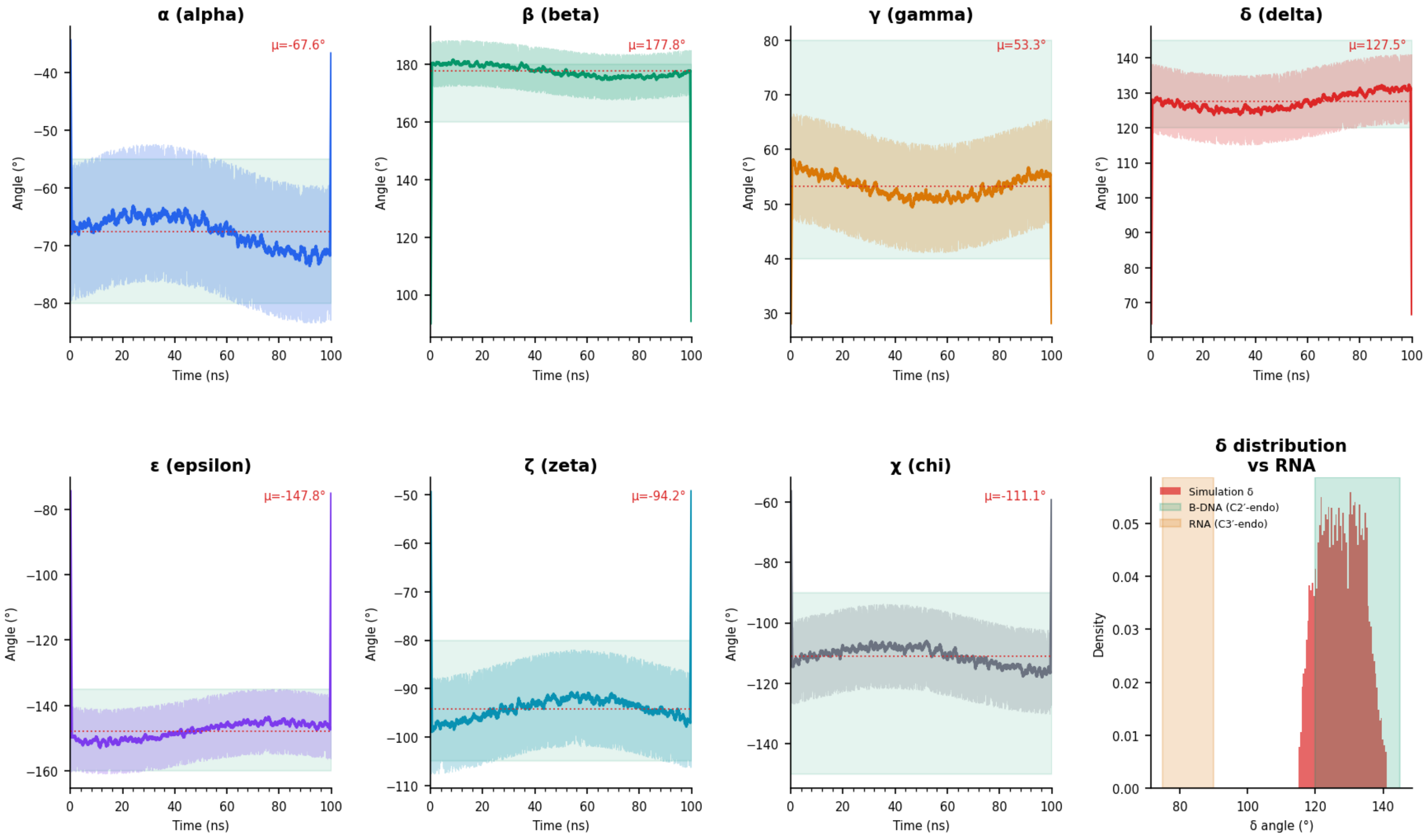


**Figure S3. Backbone conformational analysis of the isolated MDV S–IR ssDNA stem–loop during a 100 ns MD simulation.** Time-dependent profiles of the backbone torsion angles α, β, γ, δ, ε, ζ, and χ are shown. Shaded regions indicate the expected conformational ranges for canonical B-DNA. The final panel shows the distribution of the sugar-pucker parameter (δ) relative to the characteristic C2′-endo (B-DNA) and C3′-endo (RNA) conformations. The observed torsion-angle behavior and sugar-pucker distribution remained consistent with a stable DNA-like conformation throughout the simulation.

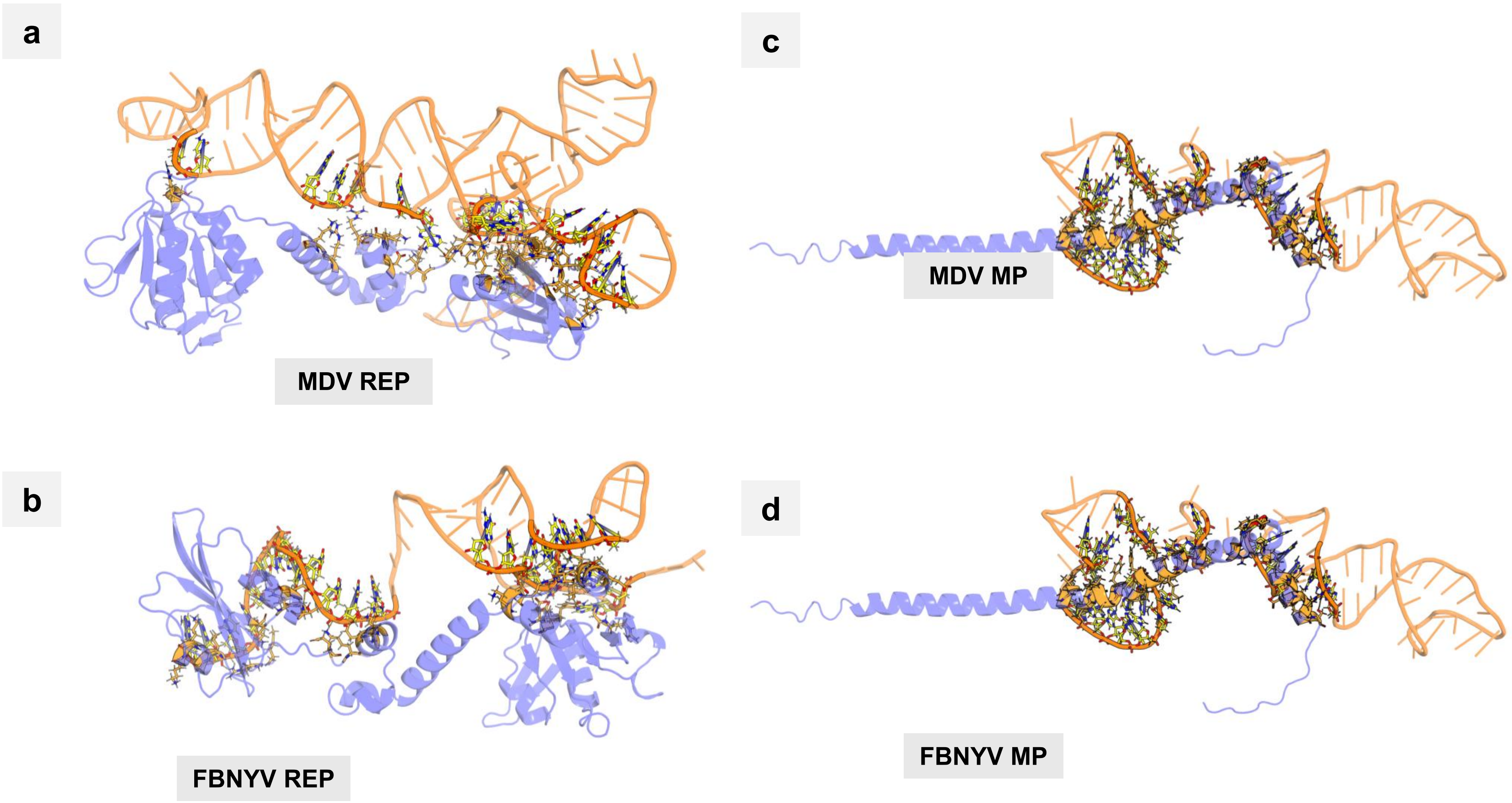


**Figure S4. Representative hydrogen bond interactions at the protein–DNA interface for all complexes.** (a) MDV Rep × MDV S–IR, (b) FBNYV Rep × MDV S–IR, (c) MDV MP × MDV S–IR, and (d) FBNYV MP × MDV S–IR. Protein structures are shown in cartoon representation (blue), and the ssDNA is shown in orange. Hydrogen bonds are depicted as dashed lines, highlighting interaction networks primarily localized around the stem–loop region of the DNA. Rep complexes exhibit a denser and more continuous hydrogen bond network compared to MP complexes, consistent with stronger and more stable protein–DNA interactions.

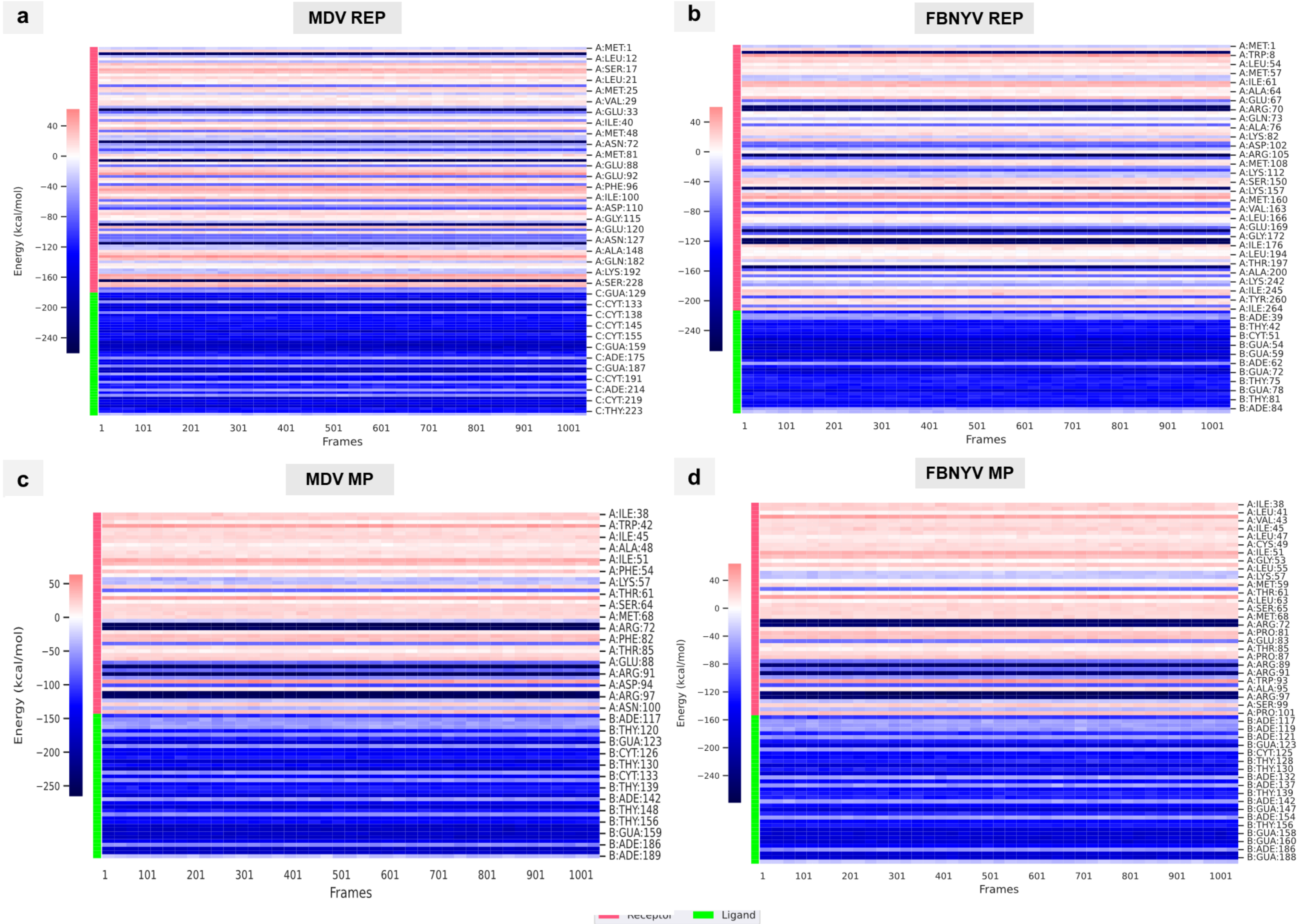


**Fig. S5. Per-residue binding free energy decomposition mapped across simulation frames for all protein–DNA complexes.** Heatmaps represent the contribution of individual residues to binding free energy (kcal/mol) over the course of the trajectory for (a) MDV Rep × MDV S–IR, (b) FBNYV Rep × MDV S–IR, (c) MDV MP × MDV S–IR, and (d) FBNYV MP × MDV S–IR. Warmer colors indicate unfavorable contributions, whereas cooler colors indicate favorable interactions. Residues belonging to the protein (receptor) and DNA (ligand) are indicated along the vertical axis. Rep complexes display more pronounced and consistently favorable contributions across multiple residues, whereas MP complexes exhibit weaker and more diffuse interaction patterns, reflecting reduced binding stability.

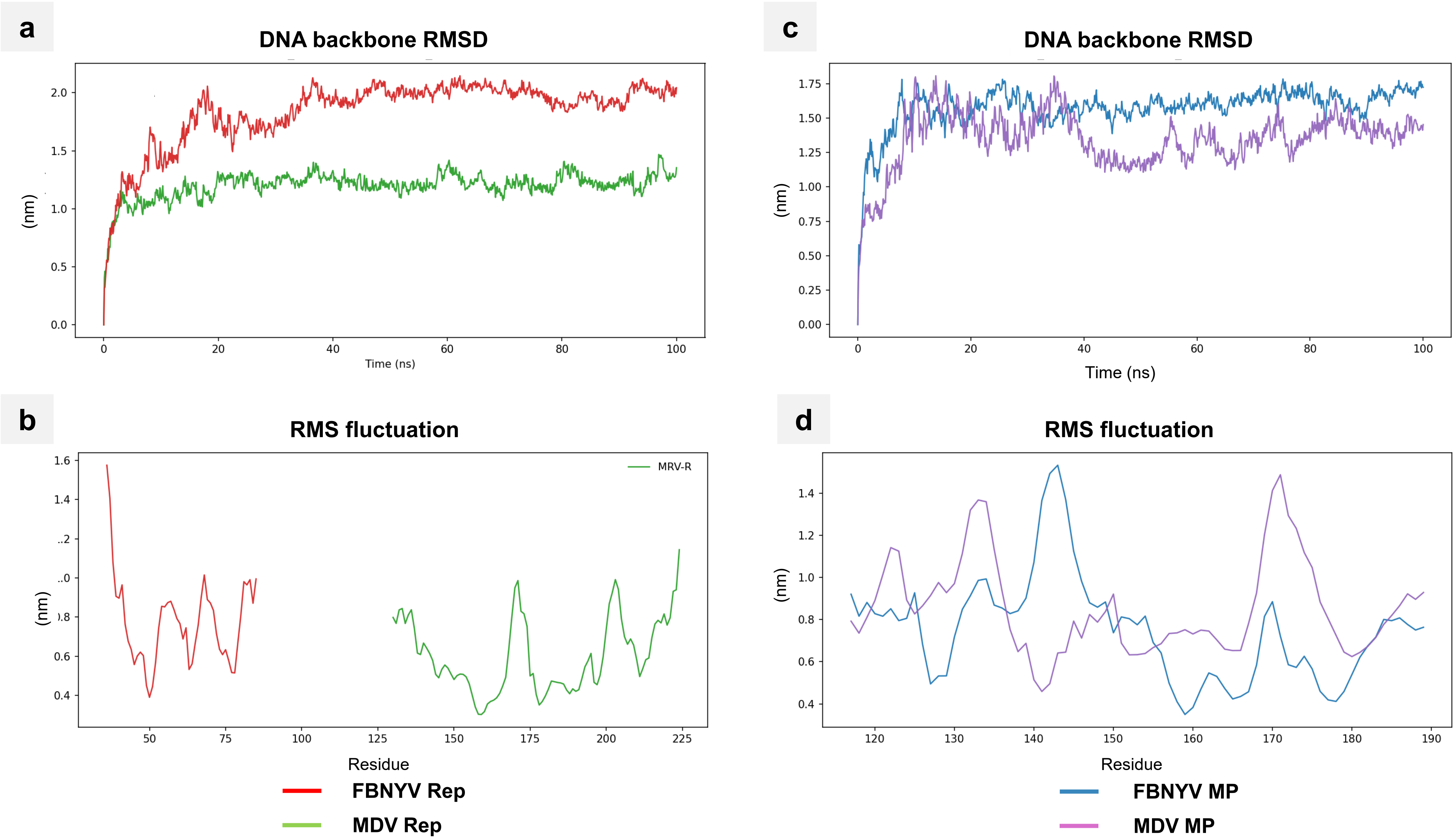


**Fig. S6. Structural stability and flexibility analysis of the DNA component in protein–DNA complexes during molecular dynamics simulations.** RMSD of the DNA backbone (phosphorus atoms) over 100 ns for Rep (a) and MP (c). RMSF profiles of DNA residues for Rep (b) and MP (d). RMSD profiles indicate initial equilibration followed by stable trajectories, while RMSF analysis highlights residue-level flexibility variations. Overall, the DNA maintains a stable conformation across all complexes, with moderate fluctuations reflecting localized structural adaptability during protein interaction.

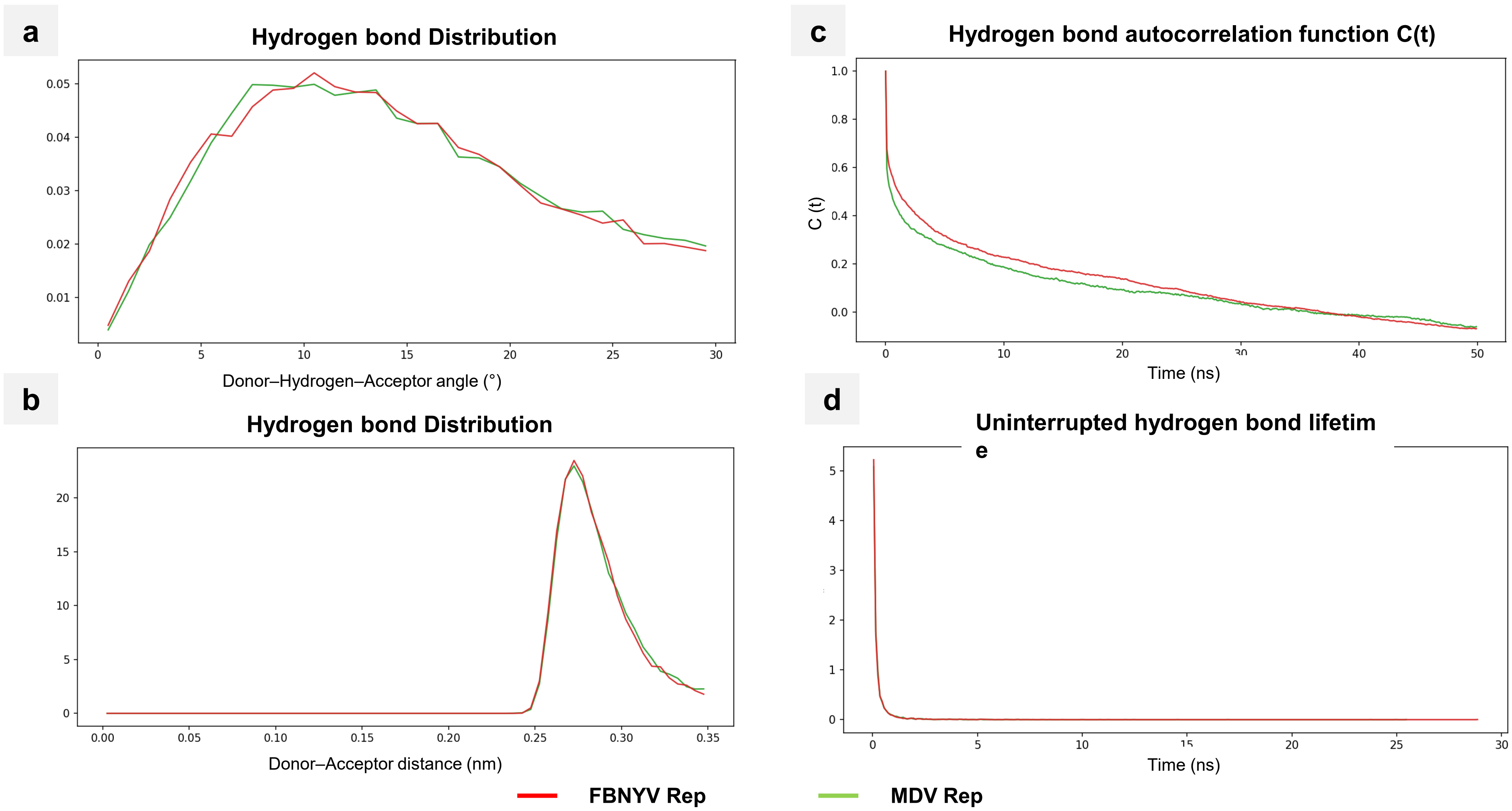


**Fig. S7. Comprehensive hydrogen bond analysis for Rep–IR complexes.** (a) Distribution of hydrogen bond donor–hydrogen–acceptor angles, showing a broad range characteristic of dynamic protein–DNA interactions. (b) Distribution of donor–acceptor distances, with a peak around ~0.27 nm consistent with standard hydrogen bond geometry. (c) Hydrogen bond autocorrelation function C(t), indicating rapid initial decay followed by a slower phase, reflecting a mixture of short-lived and persistent interactions. (d) Uninterrupted hydrogen bond lifetime analysis, demonstrating that most hydrogen bonds are transient with sub-nanosecond lifetimes. Together, these results indicate a dynamic but stable hydrogen bonding network at the protein–DNA interface.

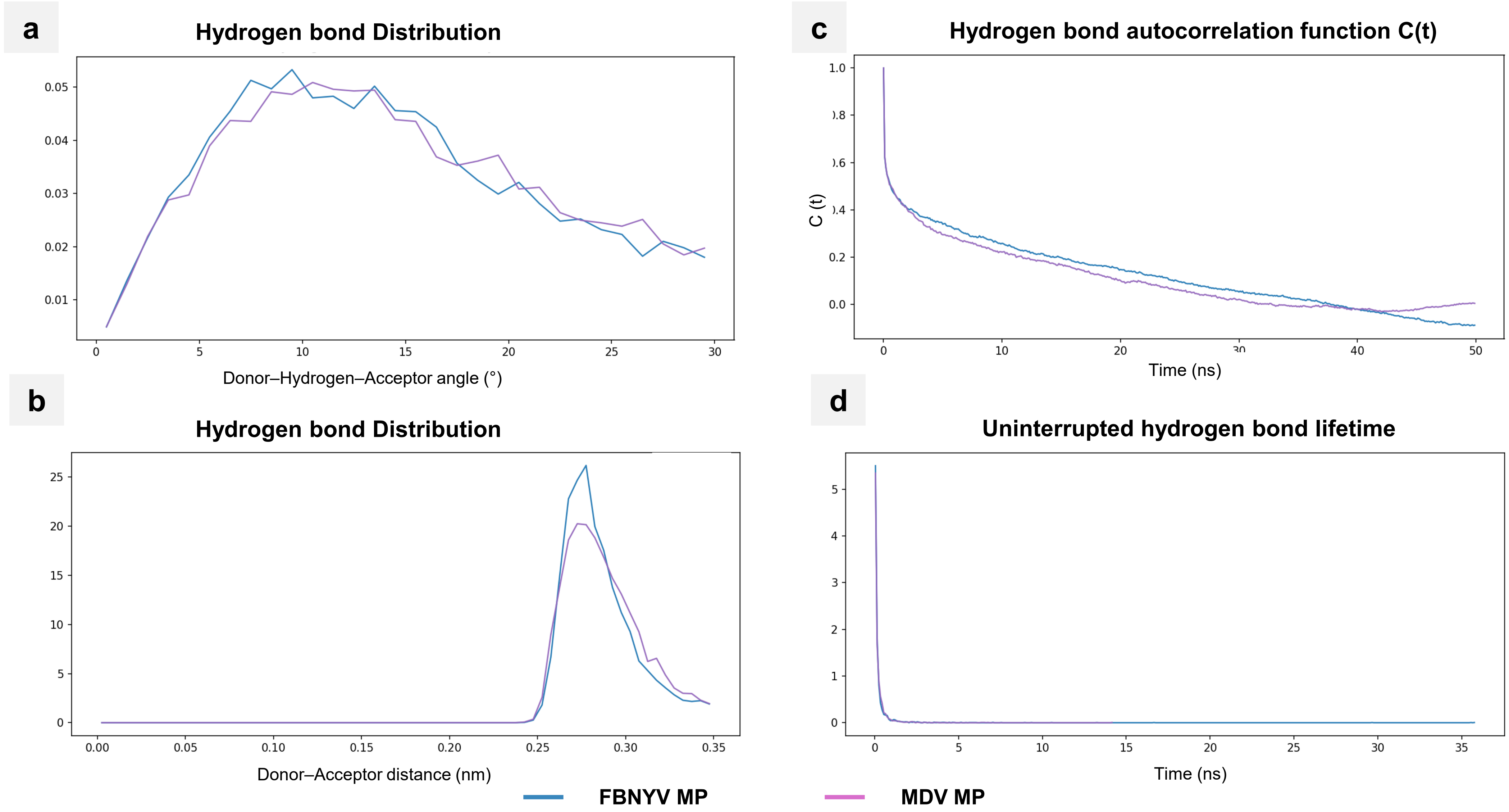


**Fig. S8. Hydrogen bond analysis for MP–IR complexes.** (a) Distribution of donor–hydrogen–acceptor angles showing a broad range characteristic of dynamic interactions. (b) Donor–acceptor distance distribution with a peak around ~0.27 nm, consistent with canonical hydrogen bond geometry. (c) Hydrogen bond autocorrelation function C(t), indicating rapid decay and reduced persistence compared to Rep systems. (d) Uninterrupted hydrogen bond lifetime analysis demonstrates predominantly short-lived interactions. Overall, MP complexes exhibit fewer and less persistent hydrogen bonds, reflecting weaker and more transient protein–DNA interactions relative to Rep complexes.
